# Genomic Prediction of Gene Bank Wheat Landraces

**DOI:** 10.1534/g3.116.029637

**Published:** 2016-04-25

**Authors:** José Crossa, Diego Jarquín, Jorge Franco, Paulino Pérez-Rodríguez, Juan Burgueño, Carolina Saint-Pierre, Prashant Vikram, Carolina Sansaloni, Cesar Petroli, Deniz Akdemir, Clay Sneller, Matthew Reynolds, Maria Tattaris, Thomas Payne, Carlos Guzman, Roberto J. Peña, Peter Wenzl, Sukhwinder Singh

**Affiliations:** * Genetic Resources Program and the Global Wheat Program, International Maize and Wheat Improvement Center (CIMMYT), 06600, Mexico, DF, Mexico; † Department of Agronomy and Horticulture, University of Nebraska-Lincoln, 321 Keim Hall, Lincoln, Nebraska 68583-0915; ‡ Departamento de Biometría, Estadística y Computación, Facultad de Agronomía, Universidad de la República (Udelar), Paysandú, Uruguay; § Colegio de Post-Graduados, Montecillos, Edo. de Mexico, 56230 Mexico; ** Department of Plant Breeding & Genetics, Cornell University, Ithaca, New York 14853; †† Department of Horticulture and Crop Science, Ohio State University, Wooster, Ohio 44691

**Keywords:** Gene bank accessions, genomic prediction, cross-validations, reference core subsets, A × E: accession × environment interaction, GenPred, shared data resources, genomic selection

## Abstract

This study examines genomic prediction within 8416 Mexican landrace accessions and 2403 Iranian landrace accessions stored in gene banks. The Mexican and Iranian collections were evaluated in separate field trials, including an optimum environment for several traits, and in two separate environments (drought, D and heat, H) for the highly heritable traits, days to heading (DTH), and days to maturity (DTM). Analyses accounting and not accounting for population structure were performed. Genomic prediction models include genotype × environment interaction (G × E). Two alternative prediction strategies were studied: (1) random cross-validation of the data in 20% training (TRN) and 80% testing (TST) (TRN20-TST80) sets, and (2) two types of core sets, “diversity” and “prediction”, including 10% and 20%, respectively, of the total collections. Accounting for population structure decreased prediction accuracy by 15–20% as compared to prediction accuracy obtained when not accounting for population structure. Accounting for population structure gave prediction accuracies for traits evaluated in one environment for TRN20-TST80 that ranged from 0.407 to 0.677 for Mexican landraces, and from 0.166 to 0.662 for Iranian landraces. Prediction accuracy of the 20% diversity core set was similar to accuracies obtained for TRN20-TST80, ranging from 0.412 to 0.654 for Mexican landraces, and from 0.182 to 0.647 for Iranian landraces. The predictive core set gave similar prediction accuracy as the diversity core set for Mexican collections, but slightly lower for Iranian collections. Prediction accuracy when incorporating G × E for DTH and DTM for Mexican landraces for TRN20-TST80 was around 0.60, which is greater than without the G × E term. For Iranian landraces, accuracies were 0.55 for the G × E model with TRN20-TST80. Results show promising prediction accuracies for potential use in germplasm enhancement and rapid introgression of exotic germplasm into elite materials.

Breeding gains have depended largely on having access to useful genetic variation in crop gene pools. Gene banks are the repositories of novel and useful genetic variation contained in a crop’s gene pool. The wheat gene bank of the International Maize and Wheat Improvement Center (CIMMYT) has access to various germplasm pools, and preserves an array of wild relatives, landraces, genetic stocks, and cultivar germplasm materials. Wheat landrace germplasm has the advantage over wild relatives of being more easily crossed with cultivated hexaploid wheat. The potential of these landraces should be harnessed for present and future wheat genetic improvement programs, and such efforts have begun (Ayala *et al.* 2013; Caballero *et al.* 2010; Guzman *et al.* 2014, 2015; Vikram *et al.* 2016). Part of the existing genetic variation in CIMMYT’s wheat and maize gene banks was characterized recently by phenotyping and genotyping thousands of accessions through the Seeds of Discovery (SeeD; http://seedsofdiscovery.org) project funded by the Mexican government through the Sustainable Modernization of Traditional Agriculture program (MasAgro; http://masagro.mx).

Evaluation and use of the genetic resources stored in gene banks can be facilitated by forming reference core sets. Initially put forward by Frankel and Brown (1984) and Brown (1989), developing core sets consists of sampling only a small proportion of all the collections of a species that will maximize allele diversity. Several authors have since studied different sampling strategies for forming “diversity” core sets using phenotypic and molecular marker data (Franco *et al.* 2006, 2010). Recently, diversity core sets of Mexican and Iranian bread wheat landraces that maximize allele diversity were prepared by using high-throughput molecular markers, for their enhanced use in wheat breeding through genome-wide association analysis (Vikram *et al.* 2016).

Rapid and precise breeding is required by crop improvement programs, and various marker-assisted methods have proven their relevance in different cereal crops. One of these methods is genomic selection (GS), which is becoming a standard approach to achieve genetic progress in plants. GS models often accurately predict the value of nonphenotyped plants, and reduce generation intervals by reducing the need for field testing progeny (Meuwissen *et al.* 2001). Further, combining methods involving high-density marker platforms with models that include genotype × environment (G × E) interactions adds power to GS models; however, Schulz-Streeck *et al.* (2013) found contrasting prediction results when using G × E. Genomic prediction models have been proposed that take into account the random effects of markers and their interaction with environments by considering Gaussian processes with covariance functions based on genetic and environmental similarities among individuals (Burgueño *et al.* 2012; Jarquín *et al.* 2014).

Although the accessions stored in gene banks represent a rich asset for breeders, alleles need to be moved from the accessions to cultivar development programs. Lengthy prebreeding programs are required to develop lines that combine favorable alleles from the germplasm bank with good agronomic performance, and thus can be used as parents in a breeding program. In recent years, genomic selection and prediction have been studied in bread wheat using only elite germplasm sets (de los Campos *et al.* 2009, 2010; Crossa *et al.* 2010; González-Camacho *et al.* 2012; Heslot *et al.* 2012; Pérez-Rodríguez *et al.* 2012; López-Cruz *et al.* 2015). To date, no study has been reported on the genomic prediction accuracy of traits measured in wheat gene bank accessions, or on the use of genomic G × E models on wheat bank accessions evaluated in different environments. In a recent study, Gorjanc *et al.* (2016) performed extensive computing simulation to evaluate germplasm enhancement schemes within the SeeD initiative for harnessing polygenic variation from maize landraces using genomic selection. Based on the simulation of various prebreeding options, Gorjanc *et al.* (2016) concluded that germplasm enhancement breeding programs can be initiated directly from landraces or landraces crossed with elite testers.

Recently, methods for selecting efficient training populations to be used in GS and prediction (Akdemir *et al.* 2015) have been developed to select a training set based on molecular marker genotyped that minimizes the predictive error variance. From the perspective of selecting a subset of individuals from the entire population, this can be considered a method for selecting a ‘prediction’ core set.

In light of the above, the objectives of this study were: (1) to examine the genomic prediction accuracy within a large number of Mexican and Iranian wheat landraces held in CIMMYT’s gene bank for several phenotypic traits; two highly heritable traits were measured in two environments [*i.e.*, days to heading (DTH), and days to maturity (DTM) evaluated in drought and heat environments], and several other traits were measured in a single optimum environment; and (2) to study two genomic prediction strategies: (i) random cross-validation schemes where 20% of the accessions form the training (TRN20) set, and 80% of the accessions comprise the testing set (TST80) (TRN20-TST80); (ii) studying the prediction accuracy of two types of reference core sets (diversity and prediction) that included 10% and 20%, respectively, of the total collections to predict the remaining 90% and 80% of the accessions, respectively.

For traits measured in a single optimum environment, we used the standard Genomic Prediction Best Linear Unbiased Predictor (GBLUP) model for the TRN20-TST80 partitions and the 10% and 20% diversity and prediction core sets. For the two traits that were measured in two environments, we used a G × E reaction norm model (Jarquín *et al.* 2014) for predicting the genetic value of 80% of the accessions for which there were no phenotypic data available in either of the two environments. For core sets, we directly predicted the 80% and 90% of the accessions that were not observed in both environments. Prediction accuracy within each collection (Mexican and Iranian) was performed accounting and not accounting for population structure.

Note that this study focuses on each collection of accessions (Mexican and Iranian separately), and that prediction results from both collections cannot be compared due to differences in population size, number of markers, and traits measured and evaluated in different field experiments. We included results of both collections in the same tables and/or figures for easy presentation, but not for comparing Mexican and Iranian landrace collections.

## Materials and Methods

We used a total of 8416 Mexican and 2403 Iranian bread wheat (*Triticum aestivum*) landrace accessions held in CIMMYT’s wheat gene bank. All of the landraces were genotyped using genotyping by sequencing (GBS) methods, and more than 40,000 SNPs were detected. The final number of markers used for each trait in both sets of landraces varied (Table 1).

**Table 1:** Phenotypic traits of Mexican and Iranian gene bank landrace collections, number of accessions, number of markers, and heritability (*h*^2^) of the trait

Trait	Number of Accessions	Number of Markers	*h*^2^
Mexican collection			
Days to heading (DTH)	8481	23,747	0.556
Days to maturity (DTM)	8481	23,747	0.554
Plant height (PHT)	8414	23,756	0.345
Grain yield per square meter (GYSM)	8142	23,740	0.339
Thousand-kernel weight (TKW)	8102	23,855	0.583
Test weight (TW)	8102	23,855	0.527
Grain hardness (GH)	7863	23,574	0.448
Grain protein (GP)	8101	23,849	0.508
SDS sedimentation (SDS)	8093	23,946	0.504
Iranian collection			
Days to heading (DTH)	2374	39,758	0.827
Days to maturity (DTM)	2374	39,758	0.822
Thousand-kernel weight (TKW)	2000	33,709	0.833
Test weight (TW)	2000	33,709	0.754
Grain hardness (GH)	2000	33,709	0.839
Grain protein (GP)	2000	33,709	0.625
Grain length (GL)	2000	33,709	0.881
SDS sedimentation (SDS)	2000	33,709	0.681
Grain width (GW)	2000	33,709	0.848
Plant height (PHT)	2000	33,709	0.434

### Phenotypic traits in Mexican and Iranian landrace data

The accessions were evaluated in the field and laboratory for several traits, and the two collections (Mexican and Iranian) were planted in different field experiments. DTH and DTM of the Mexican and Iranian wheat landraces were evaluated in field drought (D) and heat (H) experiments at CIMMYT’s experiment station near Ciudad Obregon, Sonora, northwest Mexico (27°20′ N, 109°54′ W, 38 meters above sea level), during the 2010–2011 Obregon cycle. Heat stress trials were planted in April 2011, and drought stress trials were planted in November 2010. Both heat and drought are common in wheat growing areas, and they considerably affect several important traits. The drought trial was sown during the normal wheat planting cycle and harvested in April. Plots received ∼250 mm of water through drip irrigation during the entire cropping cycle. The heat stress trial was sown on a delayed sowing date in early April (sowing date: 6–8 April; first irrigation: April 10, 2011), and harvested in July 2011. This trial was fully irrigated to keep water limitations from confounding the heat-stress results. The heat-stress trial had an average daily maximum temperature of 36.3° (min 18.1°), compared to 27.8° (min 9.0°) for the drought stress trial.

In both drought and heat stress trials, fertilization, as well as weed, disease, and pest control, were applied as necessary to minimize limitations. The Mexican and Iranian landraces were evaluated in two separate trials using an augmented grid-check field design with three randomized checks distributed along rows and columns. Plots were 0.40 m^2^. For each accession of each collection, DTH was determined as the number of days from emergence to 50% spike emergence. DTM was measured as the number of days when 50% of peduncles were completely yellow.

Other traits were measured on Mexican and Iranian landraces, but only in a single, optimum, well-irrigated environment in Cd. Obregon using an augmented grid-check field design. For Mexican accessions evaluated in the optimum environment, the other traits measured were: thousand-kernel weight (TKW), test weight (TW), grain hardness (GH), grain protein (GP), SDS sedimentation (SDS), grain yield per square meter (GYSM) and plant height (PHT) (Table 1). Traits measured on Iranian landraces only in the optimum environment in Cd. Obregon were thousand-kernel weight (TKW), test weight (TW), grain width (GW), grain hardness (GH), grain protein (GP), grain length (GL), plant height (PHT), and SDS sedimentation (SDS).

### Spatial analyses of field experiments

In agricultural field trials, accounting for major and minor sources of spatial variation in plot errors is of paramount importance (Gilmour *et al.* 1997). In this study, the raw data for all traits in each Mexican and Iranian landrace experiment were corrected for plot-to-plot variability using a separable autoregressive model fitted in the direction of the rows and the columns of the spatial coordinates, in which the data are observed on a regular grid (Gilmour *et al.* 2009). The autoregressive model is a random process that describes processes that vary in space (or time). In this autoregressive process, the value of the response trait in one experimental plot depends on the trait’s other values in spatially related plots and represents a special case of a time series problem (when one data value depends on other previously observed data values).

### Genotypic data

The DArT-Seq (Sansaloni *et al.* 2011) platform was used to generate genomic profiles of Mexican and Iranian wheat landraces. This technology combines DArT complexity reduction methods with next generation sequencing platforms, which allow scanning over 100,000 loci for DNA variation primarily targeting genetic regions. DArTseq integrates silico-DArT markers (based on SNP and methylation variation) with “traditional” SNP markers on the fragments detected in genomic representation (Carling *et al.* 2015). Two enzymes (*Pst*I and *Hpa*II) were used to create genomic representations of both populations. The samples were submitted to digestion and ligation of barcode adaptors, which allow multiplexing 96 samples in a single lane of an Illumina Hiseq2500 (Illumina Inc., San Diego, CA). More than 2,000,000 tags per sample generated up to 77 bases. A DArT P/L analytical pipeline was used to generate allele calls for SNP and silico-DArT. From a total of 40,000 markers, a set of filtering parameters was applied to select and provide high quality markers. Marker filtering was done in a trait-specific manner because some traits were not measured in all the lines; thus these lines had to be deleted from the prediction.

The number of markers used in each data set for each trait measured in Mexican and Iranian landraces are shown in Table 1. Genomic heritability (*h*^2^) (shown in Table 1) was computed as the ratio between the genetic variance due to markers over the summation of the genetic variance plus the error variance ($h^{2}=\frac{\sigma_{g}^{2}}{\sigma_{g}^{2}+\sigma_{e}^{2}}$).

### Defining 10% and 20% diversity core sets of wheat Mexican and Iranian landraces

To develop a suitable genomic selection training model, we sampled reference core sets of the Mexican and Iranian wheat landraces. Reference core sets were formed solely with genotypic marker information. Two such representative sets with 10% and 20% of the Mexican and Iranian landrace collections were selected to maintain the collections’ genetic diversity based on genetic distance: these reference core sets are named diversity core sets.

To define the reference core sets, allele frequencies were calculated; modified Rogers genetic distances (MRD; Reif *et al.* 2005) between pairs of accessions were then computed and the accessions classified into groups by the hierarchical “minimum variance within group” clustering method (Ward 1963) using the “hclust” routine from the “fastcluster” R package (R Core Team 2016). Using the MRD and the minimum variance within group strategy guarantees that the individuals within a group have a low average distance, while individuals between groups have a high average distance. Using the MRD genetic distance is justified because it satisfies the condition of being a Euclidean distance (Franco *et al.* 2005).

To define the number of groups in each set, different partitions of accessions were analyzed using the “rect.hclus” routine from R; this routine draws rectangles around the different branches of a dendrogram so that corresponding clusters are highlighted. Sample size per group was defined by average MRD distance values calculated for each cluster and the number of accessions to be obtained per cluster was defined as being proportional to the group average distance (D-method, Franco *et al.* 2005). Once sample size per group was defined, 1000 independent “candidate subsets” were selected from the collection by the stratified random sampling method using the previously defined number of accessions per group. An average MRD distance value was calculated for each of the “candidate subsets,” and the subset showing the highest average MRD value was selected as the training set.

### Defining 10% and 20% prediction core sets of wheat Mexican and Iranian landraces

Here the selection of the core set was based on the reliability measure of VanRaden (2008) that is expressed as $G_{21}{\left(G_{11}+\frac{1-h^{2}}{h^{2}}\right)}^{-1}G_{21}^{’}$, where $G_{11}$ is the genomic relationship matrix of the individuals in the training set, $G_{21}$ is the genomic relationship among individuals in the training and testing sets, and $h^{2}\;$ is the trait’s genomic heritability. This reliability measure is related to the prediction error variance (PEV), and also to the coefficient of determination.

When sample size increases, computation of that reliability measure is difficult to do in practice. A solution to this problem was recently proposed by Akdemir *et al.* (2015), who proposed including a set of individuals that form the training set (*i.e.*, individuals for phenotyping and genotyping), and a second set containing the testing sets on which the prediction model is validated. The authors proposed approximating the PEV as the objective function to be minimized by applying an efficient method that uses the first 100 principal components of the marker data. In this study, the proposed method to approximate the PEV was used to form 10% and 20% prediction core sets that will be the training populations of Mexican and Iranian landraces used to predict the remaining accessions.

### Statistical models

We used the standard GBLUP genomic prediction model for traits evaluated in a single environment, and a G × E model that extended the GBLUP theory to a reaction norm model (Jarquín *et al.* 2014) for traits DTH and DTM measured in D and H environments. Phenotypic data were obtained after correcting for the field experimental design, and doing a spatial field adjustment based on the repeated check using the spatial autoregressive model described above.

#### Genomic prediction accuracy accounting and not accounting for population structure:

Genomic accuracy is affected by population structure and/or linkage disequilibrium (LD) between markers and QTL (Daetwyler *et al.* 2015). Population structure affects genetic variation because some individuals are more related than others, or because a sample may contain individuals from different groups with similar allele frequencies within groups but with different reference allele among them at a particular locus. One way to remove this structural variability due to stratified populations would be to include the first few principal components in the analyses as fixed effects; this will decrease genomic prediction accuracy (Daetwyler *et al.* 2015).

**Figure 1:**
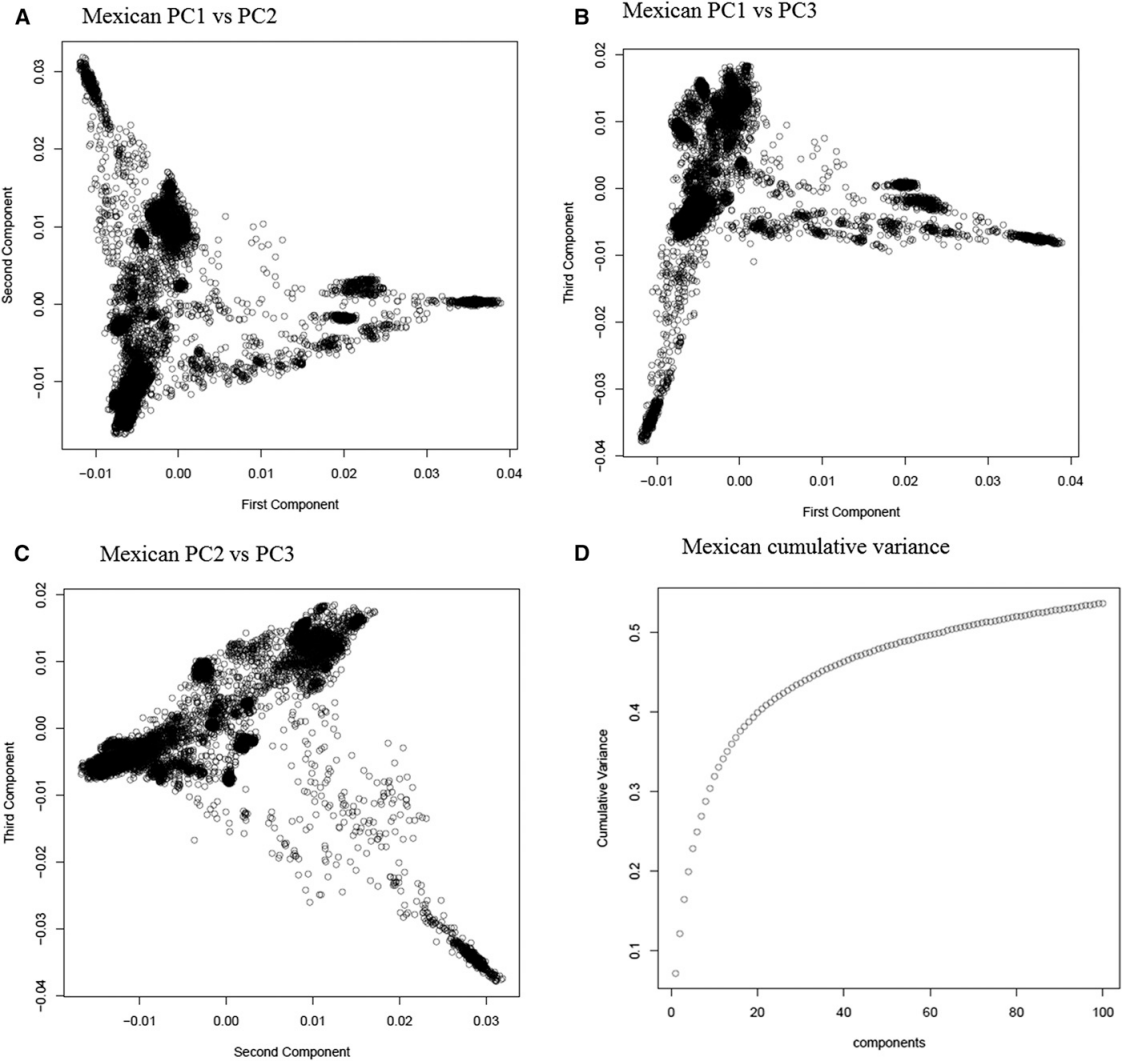

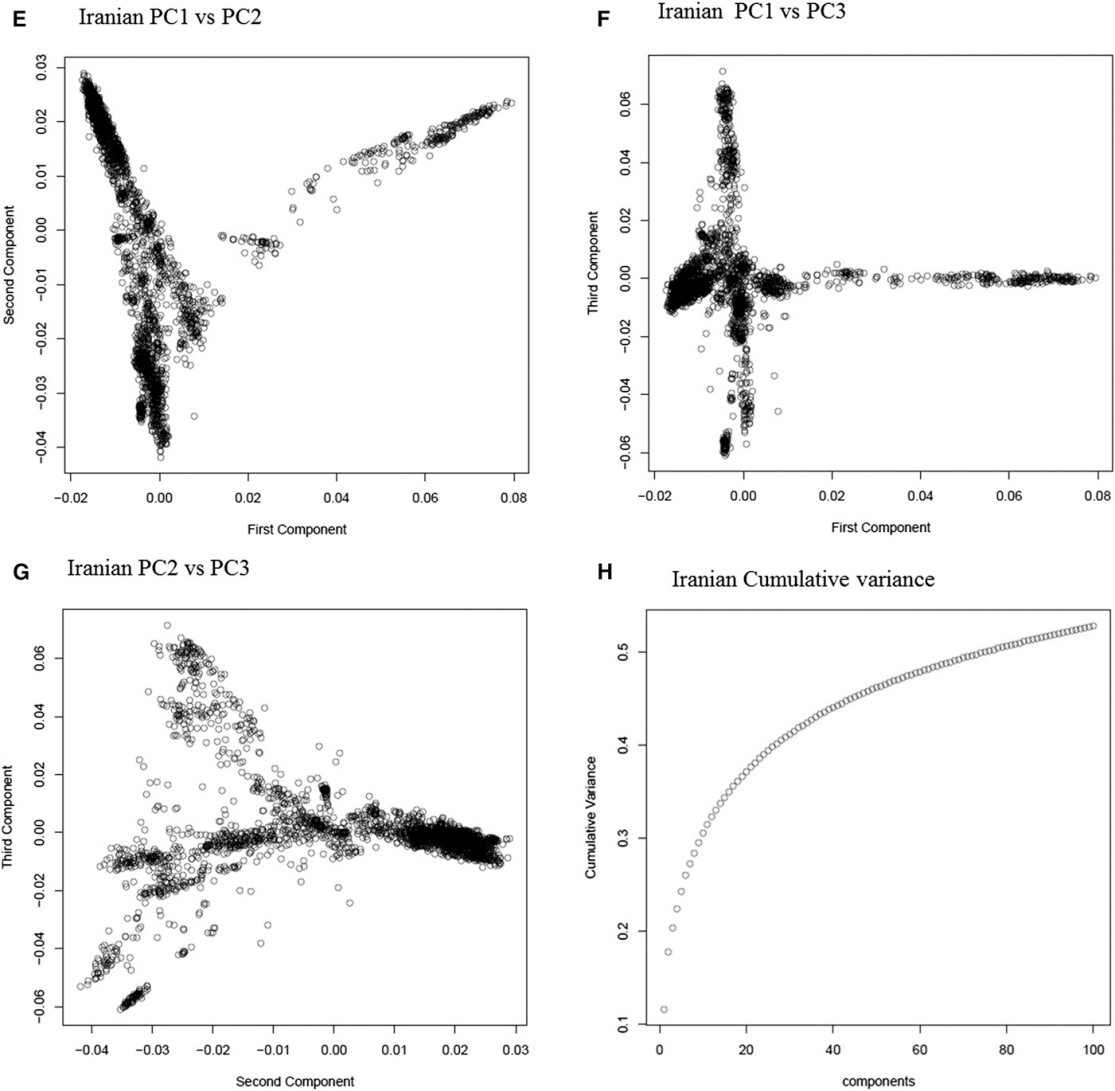
Plot of the (A) first *vs.* second principal component (PC1 *vs.* PC2) from the marker data for Mexican landraces; (B) first *vs.* third principal component (PC1 *vs.* PC3) from the marker data for Mexican landraces; (C) second *vs.* third principal component (PC2 *vs.* PC3) from the marker data for Mexican landraces; (D) cumulative variance of the various principal components; (E) first *vs.* second principal component (PC1 *vs.* PC2) from the marker data for Iranian landraces; (F) first *vs.* third principal component (PC1 *vs.* PC3) from the marker data for Iranian landraces; (G) second *vs.* third principal component (PC2 *vs.* PC3) from the marker data for Iranian landraces; (H) cumulative variance of the various principal components.

As expected, in the extensive gene bank collections of landrace accessions included in this study (Mexico and Iranian), there is a population structure. Although it is difficult to decide whether to adjust for population structure, we performed the prediction (i) accounting for population structure, and (ii) not accounting for population structure. To account for population structure, analyses were based on the relative contribution to molecular variance. The plots of the proportion of total variance explained by the eigenvalues of the genomic relationship matrix **G** for Mexican and Iranian landraces shown in Figure 1, A–H depicted a substantial population structure. The first five and nine eigenvectors for the Mexican and Iranian landraces accounted for about 25% and 30% of the total variance, respectively. We used the first five eigenvectors to adjust phenotypes for population structure. This number of eigenvectors was also suggested by Gou *et al.* (2014) in stratified maize and rice populations.

When accounting for population structure by correcting the phenotypic response variables of all the traits by the first five principal components, prediction accuracies decreased by about 15–20% (on average) as compared with analyses that did not account for population structure. Full results of the prediction accuracy of models fitted without correcting for population structure are given in the *Appendix*.

#### GBLUP single-environment model for traits measured in one environment:

We applied a single-environment model for several traits that were measured in one environment. This model regresses the phenotype vector containing the records for the response variable, $y=\left\{y_{\text{i}}\right\}$ (where *i* indexes wheat landraces accessions), on markers using a linear model of the form $\;y_{\text{i}}=\mu +\overset{p}{\underset{k=1}{\sum }}x_{\text{ik}}\beta_{\text{k}}+\;\varepsilon_{\text{i}}$, (*i* = 1,2,…,*n* accessions; *k* = 1,2,…,*p* markers) or, in matrix notation,

y=1μ+ Xβ+ε (1)

where μ is an intercept, $X=\{x_{\text{ik}}\}$ is a matrix of marker genotypes, $\beta =\{\beta_{\text{k}}\;\}$ is a vector of marker effects and ε is a vector of model residuals. The assumptions of the GBLUP model (VanRaden, 2007, 2008) are that $\beta \sim N(0,I\sigma_{\text{β}}^{\text{2}})$, and $\varepsilon \sim N\left(0,I\sigma_{\text{ε}}^{2}\right)$. Setting g=Xβ, model (1) can be represented as:

y=1μ+ g+ε (2)

with $g\sim N\left(0,G\sigma_{\text{u}}^{2}\right)$, with the entries of **G** given by $\frac{\sum_{\text{k}=1}^{\text{p}}(x_{\text{ik}}-2p_{\text{k}})(x_{\text{jk}}-2p_{\text{k}})}{\sum_{\text{k}=1}^{\text{p}}2p_{k}(1-p_{\text{k}})}$ where $p_{\text{k}}$ is the estimated allele frequency whose number of copies at the *i^th^* accession is counted in $x_{\text{ik}}$. Centering (*i.e.*, subtracting $2p_{\text{k}}$ from the genotype codes) and standardization (*i.e.*, dividing by $\overset{p}{\underset{k=1}{\sum }}2p_{\text{k}}(1-p_{\text{k}}))$ allows interpreting $\sigma_{\text{u}}^{2}=\sigma_{\text{β}}^{2}\overset{p}{\underset{k=1}{\sum }}2p_{\text{k}}(1-p_{\text{k}})$ as a genomic variance. As the number of independently segregating loci increases, the entries of the genomic relationship matrix **G** converge to twice the coefficient of parentage (or coancestry) between lines.

#### GBLUP G × E models for traits measured in two environments:

Response variables (DTH and DTM) measured in two environments (D and H) were analyzed by applying a sequence of multiplicative reaction norm models similar to that used by Jarquín *et al.* (2014) with genomic-based relationship matrices and by Pérez-Rodríguez *et al.* (2012) with pedigree-based relationship matrices. Two models (M1 and M2) included only the main effects of environment, accessions and/or genomic information. Models M3 and M4 included the main effects and different types of interactions. Several articles have used slightly different sequences of models for assessing the prediction accuracy of models including main effects and interaction terms (Zhang *et al.* 2014), depending on the structure of the main effects and interaction terms of interest. A brief description of the models considered in this study is given below.

##### Main effect model 1 (M1):

This baseline main effect model considers the response of the *j*^th^ accession in the *i*^th^ environment ($y_{\text{ij}})\;$as a function of a random effect model that accounts for only the effect of the environment ($E_{\text{i}}$), the accession ($A_{\text{j}}),$ plus a residual ($\varepsilon_{\text{j}}$):

$$y_{\text{ij}}=\mu +E_{\text{i}}+A_{\text{j}}+\varepsilon_{\text{ij}}$$ (3)

where μ is an intercept, $E_{\text{i}}\overset{iid}{\sim }N\left(0,\;\sigma_{\text{E}}^{2}\right)$ is the random effect of the *i*^th^ environment, $A_{\text{j}}\overset{iid}{\sim }N\left(0,\;\sigma_{\text{A}}^{2}\right)$ is the random effect of the *j*^th^ accession, and $\varepsilon_{\text{ij}}\overset{iid}{\sim }N(0,\sigma_{\text{ε}}^{2})$ is a model residual. Here N(⋅,⋅) stands for a normally distributed random variable, and *iid* stands for independent and identically distributed. In this model, the effects of the lines are regarded as independent; therefore, there is no borrowing of information between landrace accessions.

##### Main effect model 2 (M2):

The other main effect model adds to model (equation 3) the random effect of the *j^th^* genome $g_{\text{j}}$, which is an approximation of the true genetic value of the *j*^th^ accession (Jarquín *et al.* 2014). This approximation is given by the regression on marker covariates $g_{\text{j}}=\overset{p}{\underset{k=1}{\sum }}x_{\text{jk}}\beta_{\text{k}}$, where $x_{\text{jk}}$ is the genotype of the *j*^th^ accession at the *k*^th^ marker, and $\beta_{\text{k}}$ is the effect of the *k*^th^ marker with the assumption that $\beta_{\text{k}}\overset{iid}{\sim }N(0,\sigma_{\text{β}}^{2})$ (*k=*1,…,*p)* and $\sigma_{\text{β}}^{2}$ is the variance of the marker effects. The vector $g=\left(g_{1},\ldots ,g_{\text{J}}\right)\text{′}$ contains the genomic values of all the accessions, and is assumed to follow a multivariate normal density with zero mean and covariance matrix $Cov\left(g\right)=G\sigma_{\text{g}}^{2}$, where **G** is the genomic relationship matrix and $\sigma_{\text{g}}^{2}$ is the genomic variance, which is proportional to $\sigma_{\text{β}}^{2}$ ($\sigma_{\text{g}}^{2}\propto \sigma_{\text{β}}^{2}$). Therefore, model (3) becomes

$$y_{\text{ij}}=\mu +E_{\text{i}}+A_{\text{j}}+g_{\text{j}}+\varepsilon_{\text{ij}}\;$$ (4)

where the vector of random effects is assumed $g\sim N(0,G\sigma_{\text{g}}^{2})\;$, $E_{\text{i}}\overset{iid}{\sim }N\left(0,\;\sigma_{\text{E}}^{2}\right)$, $\;A_{\text{j}}\overset{iid}{\sim }N\left(0,\;\sigma_{\text{A}}^{2}\right)$, and $\varepsilon_{\text{ij}}$ $\overset{iid}{\sim }N(0,\sigma_{\text{ε}}^{2}).\;$ The random effects **$g=\left(g_{1},\ldots ,g_{\text{J}}\right)\text{′}$** are correlated such that model (4) allows borrowing information across $A_{\text{j}}$ accessions; thus predicting accession performance in environments where the lines were not observed is possible. As previously mentioned, $g_{\text{j}}$ approximates the true genetic values of the $A_{\text{j}}$ accession.

##### Main effect and interaction model 3 (M3):

In this study, a Gaussian process with a covariance function structured based on a reaction norm model is used to model the interaction between markers and environments. Jarquín *et al.* (2014) showed that the covariance structure is the Hadamard product of two covariance structures, one describing the relationships between lines based on genetic information, and the other relating the environments. Genetic similarity could be based on a genomic relationship matrix or on a pedigree relationship matrix; environmental similarities could be approximated by using environmental covariates (when available) (Jarquín *et al.* 2014).

The response of the trait is measured in the *j*^th^ accession in the *i*^th^ environments ($y_{\text{ij}}$) and explained by including the random main effects of model (2), $E_{\text{i}}$, $A_{\text{j}}$, and $g_{\text{j}}$, plus the random effects of the interaction between the *i*^th^ environment ($E_{\text{i}})\;$and the *j*^th^ genomic ($g_{\text{j}}$) $Eg_{\text{ij}}$. This model is obtained by extending model 2 (Equation 4) to introduce a new random effect due to interaction ($Eg_{\text{ij}}$). Thus, predictions can be obtained with the model:

$$y_{\text{ij}}=\mu +E_{\text{i}}+A_{\text{j}}+g_{\text{j}}+Eg_{\text{ij}}+\;\varepsilon_{\text{ij}}$$ (5)

where $E_{\text{i}}$, $A_{\text{j}}$ and $g_{\text{j}}$ have already been defined, $\mathrm{Eg}\sim N\left(0,\left(Z_{\text{g}}GZ_{\text{g}}^{\prime }\right){}^{\circ}\left(Z_{\text{E}}Z_{\text{E}}^{\prime }\right)\sigma_{\text{Eg}}^{2}\right)$ is the genome’s interaction with the environment, where $Z_{g}$ is the incidence matrix for the effects of the genetic values of the genotypes, $\sigma_{\mathrm{Eg}}^{2}$ is the variance component of Eg and ‘∘’ stands for the Hadamard product between two matrices. Matrix $Z_{E}$ is the incidence matrix for environments. Note that in Equation (5), the interaction term $Eg_{\text{ij }}$ approximates the interaction of the $g_{\text{j }}$accession with the *i*^th^ environment ($E_{\text{i}}$).

As discussed in Jarquín *et al.* (2014) and Pérez-Rodríguez and de los Campos (2014), the model of Equation (5) uses the covariance patterns induced by a bilinear reaction norm where the intercepts are implicitly accounted for by the main effects of accessions and the environments, while the slopes are implicitly modeled by the interaction term. The intercepts and the slopes are treated as independent.

##### Main effect and interaction model 4 (M4):

Model 4 is similar to model 3 but includes the interaction of the *j*^th^ accession with the *i*^th^ environment, $EA_{\text{ij}}$, such that

$$y_{\text{ij}}=\mu +E_{\text{i}}+A_{\text{j}}+g_{\text{j}}+EA_{\text{ij}}+Eg_{\text{ij}}+\;\varepsilon_{\text{ij}}$$ (6)

where the term $EA_{\text{ij}}$ denotes the interaction of the *j*^th^ accession in the *i*^th^ environment with $\mathrm{EA}\sim N\left(0,\left(Z_{\text{A}}IZ_{\text{A}}^{\prime }\right){}^{\circ}\left(Z_{\text{E}}Z_{\text{E}}^{\prime }\right)\sigma_{\text{EA}}^{2}\right),$ where $Z_{A}$ and $Z_{E}$ are the incidence matrices for accessions and environments, respectively, and $\sigma_{\mathrm{EA}}^{2}$ is the variance component of $EA_{\mathrm{ij}}$.

#### Model prediction using random cross-validation:

The models were fitted in a cross-validation setting to estimate prediction accuracy. For traits evaluated in one environment using the single-environment GBLUP model, we made 30 random cross-validation partitions with 20% of all accessions in the training set (TRN20) and 80% of the accessions in the testing set (TST80).

For traits DTH and DTM measured in two environments (D and H) using the G × E model, we followed Burgueño *et al.* (2012) and Jarquín *et al.* (2014), and considered the prediction of the performance of Mexico and Iranian accessions that have not been evaluated in any field trials (CV1). The CV1 partition was obtained by assigning accessions to folds; hence, when the phenotype of an accession is predicted, the corresponding training set contains no record of this accession. Depending on the trait, for the Mexican collections about 7380 (90%) and 6560 (80%) accessions were predicted, and for the Iranian collection about 1800 (90%) and 1600 (80%) accessions were predicted. For the random cross-validation, a TRN20-TST20 scheme was employed with 30 random partitions. For the 10% and 20% diversity and prediction core sets, the prediction was directly computed for the specific core type and size.

#### Model prediction using 10% and 20% diversity and prediction reference core sets:

The other prediction assessment problems we studied were those posed by using 10% and 20% reference core sets as training populations to predict the remaining 90% and 80% of the Mexican and Iranian landraces, respectively, for the core sets based on diversity or core sets based on prediction. For these prediction problems, the correlation reported for traits DTH and DTH measured in D and H environments is that obtained between the predicted values from the four G × E models, and the observed DTH and DTM values in D and H environments and across both environments. For traits measured in a single environment, the reported correlation is that obtained between the predicted values computed using GBLUP trained with 10% and 20% of the two types of reference core sets and the observed values for 90% and 80% of Mexican and Iranian landraces.

### Software

The models described above were all fitted using the BGLR R-package (Pérez-Rodríguez and de los Campos 2014; de los Campos and Pérez-Rodríguez 2014). This package can handle both molecular marker and pedigree data in parametric and semi-parametric contexts, and allows including different numbers of random effects with user-defined covariance matrices.

### Data availability

The complete phenotypic and genotypic data sets for Mexican and Iranian landraces can be downloaded from the link: http://genomics.cimmyt.org/mexican_iranian/traverse/. The Iranian directory contains the following data sets: G.RData, dth_dtm_cores.RDta, standarizedData_dth_dtm.RData, and the standarizedData_univariate.RData. The Mexican directory has one root directory, Toshare, that includes two sub-directories, G×E.Data and Univariate; the G×EData sub-directory has two files, standarizedData_dth_dtm.RData and standarizedData_core.RData, whereas the Univariate sub-directory has two files: Cores and data.Univariate.

## Results

### Traits evaluated in a single environment

#### Prediction accuracy for random cross-validation (TRN20-TST80):

Genomic heritabilities of the traits ($h^{2})$ were substantially higher for the Iranian landraces than for the Mexican landraces (Table 1), but this varies depending on the trait. For both collections, the prediction accuracies of the GBLUP model trained in one single optimum environment under random cross-validation TRN20-TST80, and adjusted for population structure, produced correlations of around 0.40-0.65 (except for PHT, 0.166, for the Iranian collection) (Table 2). For Mexican landraces, TRN20-TST80 correlations ranged from 0.407 (PHT) to 0.677 (TKW), whereas for Iranian landraces, prediction accuracies for TRN20-TST80 ranged from 0.166 (PHT) to 0.662 (GL). When not accounting for population structure, prediction accuracy increased and ranged from 0.451 (PHT) to 0.767 (TKW) for Mexican accessions and from 0.260 (PHT) to 0.688 (GW) for Iranian accessions (Table A1, *Appendix*).

**Table 2:** Accounting for population structure

Trait	TRN20-TST80	10% Diversity Core	20% Diversity Core	10% Prediction Core	20% Prediction Core
Mexican landraces					
Plant height (PHT)	0.407 (0.006)	0.359	0.412	0.353	0.405
Thousand-kernel weight (TKW)	0.677 (0.007)	0.644	0.654	0.652	0.663
Test weight (TW)	0.498 (0.008)	0.457	0.478	0.462	0.497
Grain hardness (GH)	0.458 (0.008)	0.404	0.450	0.420	0.458
Grain protein (GP)	0.516 (0.009)	0.471	0.497	0.461	0.512
SDS sedimentation (SDS)	0.571 (0.007)	0.542	0.539	0.4531	0.553
Grain yield per square meter (GYSM)	0.460 (0.006)	0.434	0.451	0.422	0.451
Iranian landraces					
Plant height (PHT)	0.166 (0.027)	0.112	0.182	0.141	0.154
Thousand-kernel weight (TKW)	0.519 (0.017)	0.463	0.468	0.445	0.475
Test weight (TW)	0.437 (0.020)	0.392	0.399	0.391	0.379
Grain hardness (GH)	0.528 (0.017)	0.447	0.520	0.386	0.463
Grain protein (GP)	0.412 (0.023)	0.417	0.408	0.385	0.386
Grain length (GL)	0.662 (0.016)	0.593	0.647	0.612	0.628
Grain width (GW)	0.502 (0.019)	0.417	0.475	0.419	0.443
SDS sedimentation (SDS)	0.390 (0.021)	0.352	0.377	0.305	0.369

Mean correlation between predicted and observed values across 30 random cross-validation partitions (SD in parentheses) for a training set of 20% (TRN20) and a testing set of 80% (TST80), of the total Mexican and Iranian collections for several traits measured in a single environment using the GBLUP model. Correlation using 10% diversity and prediction cores, and 20% diversity and prediction cores as training sets to predict the remaining 90% and 80% of the collections.

#### Prediction accuracy for the 10% and 20% diversity and prediction reference core sets:

The prediction accuracies of the GBLUP model trained with 10% and 20% diversity, and prediction core sets of the Mexican and Iranian collections are shown in Table 2. It is interesting to note that the correlations between observed and predicted values for the 20% core size (either diversity and/or prediction cores) of the Mexican landraces were slightly lower than for the random cross-validation TRN20-TST80 partition of the entire population (an exception was PHT, 20% diversity core). For example, for the Mexican collection, grain yield per square meter (GYSM) was predicted by TRN20-TST80 with an accuracy of 0.460, whereas the diversity and prediction cores sizes predicted GYSM within a range of 0.422 and 0.451 (Table 2). Trait TKW was predicted by the 10% and 20% diversity and prediction core sets with accuracies ranging from 0.644 to 0.663, whereas partition TR20-TST80 predicted this trait with an accuracy of 0.677. Interestingly, prediction accuracies of the 20% diverse and prediction core sets were only slightly higher than accuracies of the 10% diverse and prediction core sets and very similar to those achieved by random cross-validation TRN20-TST80. Therefore, for the Mexican collections, prediction accuracies from TRS20-TST80, and 10% and 20% diversity and prediction core sets were similar.

Regarding the prediction accuracy of the Iranian reference core sets, the correlations for TRN20-TST80 ranged from relatively high for GL (0.662) to low for PHT (0.116) (Table 2). The prediction accuracy for the 10% diversity core ranged from 0.112 (PHT) to 0.593 (GL), whereas for the 20% diversity core, accuracies ranged from 0.182 (PHT) to 0.647 (GL). However, for the Iranian collection, the 10% and 20% diversity and prediction core sizes tended to have slightly lower prediction accuracy than those observed in the Mexican collection (Table 2); for some traits, the prediction core gave lower accuracy than the diversity core for both sizes (10% and 20%).

The correlations increased when not accounting for population structure (Table A1, *Appendix*) in the TRN20-TST80, 10%, and 20% diversity and prediction core subsets for single environment traits. Prediction accuracies increased up to 0.767 for TKW in Mexican landraces, and up to 0.688 for GW. In general, the 10% and 20% diversity core sets achieved prediction accuracies similar to those of the TRN20-TST80 random partition. However, the 10% and 20% prediction cores did not outperform the diversity core sets or the cross-validation TRN20-TST80.

### Traits evaluated in drought and heat environments

#### Descriptive statistics:

**Figure 2:**
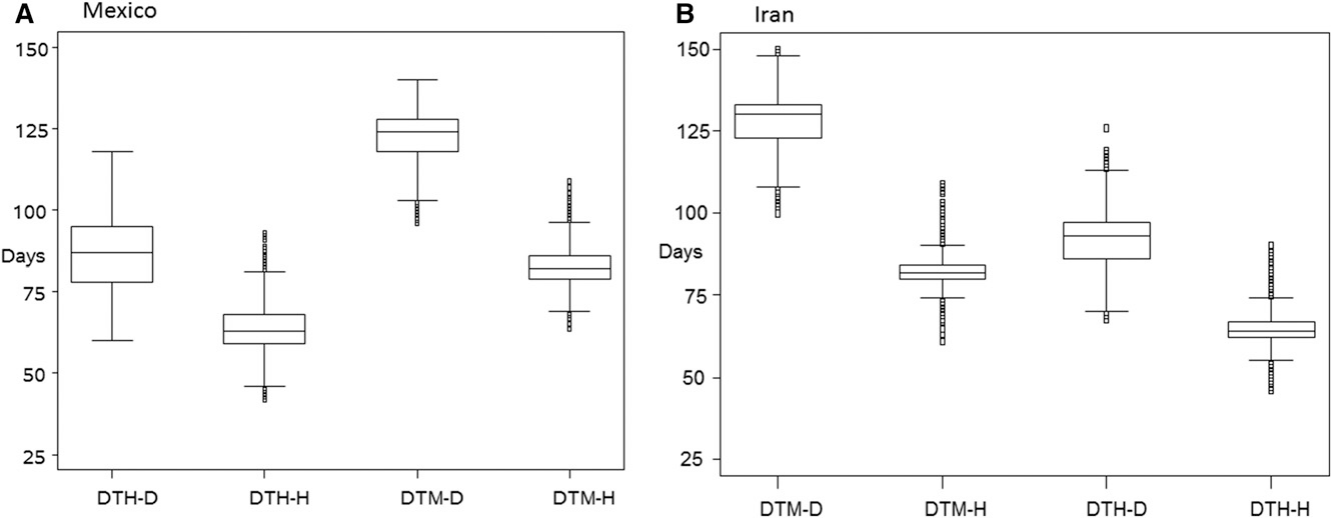
Box-plot of days to heading (DTH) and days to maturity (DTM) measured in drought (D) and heat (H) environments for (A) Mexican landraces and (B) Iranian landraces.

Box-plots of traits DTH and DTM in D and H environments are depicted in Figure 2, A and B for Mexican and Iranian landraces, respectively. Mexican and Iranian landraces in environment D planted in November headed and matured later than the ones in environment H, which were planted in April. For Mexican landraces, the range in DTM-D and DTM-H was large, ranging from 80 days (DTM-H) to 125 days (DTM-D); similar differences were found in Iranian landraces for DTM-D and DTM-H. In both sets of landraces, traits DTH-D and DTH-H tended to have a smaller range of values than DTM-D and DTM-H.

#### Sample phenotypic correlations:

Sample phenotypic correlations among the four trait-environment combinations for Mexican and Iranian landraces show that the correlations among traits (DTH-D, DTH-H, DTM-D, and DTM-H) for Mexican landraces are high, ranging from 0.5919 (for DTH-D *vs.* DTH-H) to 0.8276 (for DTH-H *vs.* DTM-H) (data not shown). For Iranian landraces, sample phenotypic correlations among the four trait-environment combinations are lower than those obtained for Mexican landraces, ranging from 0.3459 (for DTH-D *vs.* DTM-H) to 0.7939 (for DTH-D *vs.* DTM-D) (data not shown).

#### Variance component estimates:

Table 3 gives estimates of the variance components of the four models (M1–M4) for the full data sets of Mexican and Iranian landraces, for each of the traits (DTH and DTM) across environments D and H. The variance of the accessions (A) captures the difference between the accessions. The genomic variance (G) accounts for only the genetic effects captured by markers. Similarly, the interaction variance component of G × E (genomic × environment) quantifies the interaction between the genomic effects and the environments.

**Table 3:** Accounting for population structure in Mexican and Iranian landraces

	Estimated Variance Component						Percentage of Within-Environment Variance				
	E	A	G	A × E	G × E	Res.	A	G	A × E	G × E	Res.
Mexican landraces											
	Days to Heading										
M1:E + A	1.137	0.025				0.038	39.43				60.56
M2:E + A + G	0.814	0.004	0.015			0.034	8.23	27.94			63.81
M3:E + A + G + G × E	0.690	0.006	0.013		0.013	0.017	13.34	26.48		26.60	33.56
M4:E + A + G + G × E + A × E	0.635	0.006	0.014	0.005	0.012	0.012	12.06	28.15	10.80	23.85	25.12
	Days to Maturity										
M1:E + A	1.075	0.124				0.120	50.83				49.16
M2:E + A + G	0.747	0.014	0.078			0.116	6.84	37.38			55.76
M3:E + A + G + G × E	0.622	0.037	0.066		0.041	0.052	18.81	33.65		20.96	26.56
M4:E + A + G + G × E + A × E	0.566	0.035	0.069	0.015	0.038	0.039	18.04	35.01	7.72	19.58	19.63
Iranian landraces											
	Days to Heading										
M1:E + A	1.122	0.046				0.139	24.92				75.08
M2:E + A + G	0.816	0.012	0.042			0.12	7.13	23.92			68.95
M3:E + A + G + G × E	0.702	0.014	0.031		0.067	0.044	9.06	19.94		42.77	28.23
M4:E + A + G + G × E + A × E	0.595	0.013	0.034	0.016	0.059	0.036	8.02	21.43	10.04	37.56	22.95
	Days to Maturity										
M1:E + A	1.103	0.03				0.041	42.26				57.74
M2:E + A + G	0.853	0.008	0.021			0.037	12.11	32.05			55.84
M3:E + A + G + G × E	0.733	0.008	0.019		0.018	0.015	13.62	31.65		30.02	24.71
M4:E + A + G + G × E + A × E	0.637	0.007	0.019	0.006	0.015	0.013	11.96	32.26	9.54	24.72	21.51

Estimated variance components for four models (M1–M4) and percentage of within-environment variance accounted for by each random effect of the corresponding model using the full data for two traits, days to heading (DTH) and days to maturity (DTM). E, environment; A, accession; G, genomic (marker); A × E, accession × environment; G × E, genomic × environment; Res. residual.

The aim is to study the ability of the different models to predict the performance of the nonphenotyped accession within each environment, after accounting for the odd term environmental mean effect (Jarquín *et al.* 2014). Therefore, the proportional contribution of each random effect to within-environment variance is expressed relative to the total variance corrected by the variance due to the main effects of the environments.

##### Mexican landraces:

The genomic (G) random main effect, the G × E variances, and the residual variance explained the largest proportion of DTH and DTM variances within environments. The G main effect explained up to 28.15% (M4) of the variance for trait DTH, and up to 37.38% (M2) for trait DTM (Table 3). The residual variances also explained a relatively large proportion—33.56% for DTH, and 26.56% for DTM (both for M3)—of the within-environment variance for both traits.

The estimated variance due to accession (A) for all traits and models was much lower than the variance associated with G, suggesting that genomic markers are able to capture a sizable proportion of the total variability due to the main effects of accessions. Estimates from models M3 and M4 for DTH show that roughly 26–28% of within-environment variability can be explained by the main effects of markers (G), 24–26% by G × E interaction terms, and 25–34% by residuals (unaccounted factors). For DTM, the variance components of models M3 and M4 show that 33.65–35.01% of within-environment variability can be explained by main effects of markers, 19.58–20.96% by G × E, and 19.63–26.56% by residuals (Table 3). For both traits, the proportion of within-environment variation that is explained by G × E is not negligible and indicates the importance of considering such interactions in models for genomic-enabled prediction.

##### Iranian landraces:

The magnitudes of the G variance component for models M3 and M4 were similar, and explained 19.94% (model M3) of the within-environment variance for DTH and 31.65% (M3) for DTM. The G × E and residual variances also explained a large proportion of the within-environment variances for both traits (Table 3). These results suggest that markers are able to capture a sizable proportion of the variability due to the main effects of accessions. For both traits, the G × E component explained a sizable percentage of the within-environment variance.

Estimates from model M3 for trait DTH in Iranian landraces indicate that roughly 20% of the within-environment variability can be explained by main effects of markers (G), and 42.77% by interaction terms (G × E), with a residual explaining 28.23%. For trait DTM, the variance components of model M3 indicated that 31.65% of the within-environment variability can be explained by main effects of markers, 30% by G × E interaction terms, and 24.71% by residuals (Table 3). Similar to the Mexican landraces, for DTH and DTM, the proportion of within-environment variation that is explained by G × E is not negligible, and indicates the importance of considering such interactions in models for genomic-enabled prediction.

Estimates of variance components for analyses not accounting for population structure are shown in Table A2 (*Appendix*). The variance components do not change much when not accounting for population structure *vs.* variance component estimates when accounting for population structure (Table 3).

#### Assessing model prediction accuracy by random cross-validation (TRN20-TST80):

##### Prediction of Mexican landraces across environments:

The average GS accuracy of the four models for TRS20-TSN80 for both traits clearly shows that models M3 and M4 gave the highest accuracy across both environments (Table 4), where prediction accuracies of models M3 and M4 were almost equal for traits DTH and DTM (*i.e.*, 0.59–0.60). This was expected due to the small variance component of A and A × E as compared with the variances of G and G × E (Table 3). When not accounting for population structure, prediction accuracies of M3 and M4 models reached 0.74–0.76 for DTHA and DTM for TRN20-TST80 (Table A3, *Appendix*).

**Table 4:** Accounting for population structure in Mexican and Iranian landraces

Trait	Model*^a^*	TRN20-TST80	10% Diversity Core	20% Diversity Core	10% Prediction Core	20% Prediction Core
Mexican collection						
DTH	M1:E + A	0.002 (0.009)	−0.005	0.004	−0.005	−0.001
	M2:E + A + G	0.508 (0.005)	0.461	0.489	0.477	0.503
	M3:E + A + G + G × E	0.599 (0.004)	0.559	0.580	0.565	0.597
	M4:E + A + G + G × E + A × E	0.600 (0.004)	0.555	0.579	0.568	0.597
DTM	M1:E + A	0.001 (0.000)	−0.008	0.003	0.002	0.003
	M2:E + A + G	0.527 (0.005)	0.482	0.511	0.484	0.513
	M3:E + A + G + G × E	0.596 (0.004)	0.558	0.584	0.553	0.586
	M4:E + A + G + G × E + A × E	0.596 (0.004)	0.558	0.581	0.558	0.584
Iranian collection						
DTH	M1:E + A	0.001 (0.023)	0.010	−0.014	0.005	−0.003
	M2:E + A + G	0.403 (0.035)	0.344	0.389	0.389	0.397
	M3:E + A + G + G × E	0.552 (0.033)	0.504	0.551	0.511	0.544
	M4:E + A + G + G × E + A × E	0.551 (0.034)	0.496	0.545	0.514	0.548
DTM	M1:E + A	0.000 (0.021)	0.013	0.003	−0.009	0.004
	M2:E + A + G	0.450 (0.052)	0.371	0.450	0.400	0.419
	M3:E + A + G + G × E	0.551 (0.029)	0.493	0.551	0.502	0.519
	M4:E + A + G + G × E + A × E	0.548 (0.026)	0.485	0.542	0.506	0.525

Mean correlation across 30 random partitions between observed and predicted values of four models for two traits, days to heading (DTH) and days to maturity (DTM), across two environments (their standard deviation, SD), for 20% training (TRN20) and 80% testing (TST80) sets of the total number of accessions in the Mexican and Iranian collections for four models (M1–M4). Correlations between observed and predictive values for 10% and 20% diversity and prediction core sets.

a Models: E, Environment; A, accession; G, genomic relationship; A × E, accession × environment interaction; G × E, genomic × environment interaction.

##### Prediction of Iranian landraces across environments:

The average GS accuracy of the four models for cross-validations TRN20-TST80 for both traits across environments are reported in Table 4. Results show that, in general, correlation values were lower than those achieved for Mexican landraces for all trait-environment combinations. As with the Mexican landraces, models M3 and M4 gave the highest accuracy for both traits across both environments. Prediction accuracies for traits DTM and DTH were similar. Models M3 and M4 reached up to 0.551 and 0.548 prediction accuracy, respectively, for DTM and DTM under partition TRN20-TST80. Without accounting for population structure, prediction accuracies of M3 and M4 models reached 0.58–0.60 for DTH and DTM under cross-validation TRN20-TST80 (Table A3, *Appendix*).

#### Assessing model prediction accuracy by 10% and 20% diversity and prediction core sets:

##### Mexican landraces across environments:

Models M3 and M4 gave the best prediction accuracy across environments for DTH and DTM for the 10% and 20% diversity and prediction reference core sets. When the 10% and 20% diversity and prediction core sets of Mexican landraces were used to predict the remaining 90% and 80% of the Mexican accessions, correlations using models M3 and M4 for DTH and DTM ranged from 0.555 (10% diversity core DTH) to 0.584 (20% prediction core DTM) (Table 4). The accuracy obtained using the diversity core sets and the prediction core sets were very similar.

On average across environments, there is a slight decrease in prediction accuracy (about 4%) when the size of the core decreases from 20 to 10%. Also, the prediction accuracy of the 20% diversity and prediction core sets for M3 and M4 (0.58) were similar to the prediction accuracies obtained from random cross-validation TRN20-TST80 across environments (around 0.596). These results confirmed that the 20% diverse and prediction core sizes produced good accuracy when predicting the remaining 80% of Mexican landraces. For the Mexican landraces, a clear increase in prediction accuracy of models M3 and M4 was achieved when not accounting for population structure, with prediction accuracies ranging from 0.717 to 0.758 for both sizes of diversity and prediction cores (Table A3, *Appendix*).

##### Iranian landraces across environments:

Models M3 and M4 had the highest prediction accuracies in all cases, *i.e.*, TRN20-TST80, 10% and 20% diversity and prediction core sets (Table 4). For the 10% prediction core set, model M3 gave correlations of 0.5111 and 0.5021 for DTH and DTM, respectively, whereas for the 20% prediction core set, these correlations were 0.5446 and 0.5194, respectively. Prediction accuracies of the 10% and 20% diversity and prediction core sets were similar to each other and to those achieved by TRN20-TST80.

The prediction accuracies of the 20% diversity core set of Iranian landraces observed in Table 4 for models M3 and M4 are at a similar level (0.55) as the prediction accuracies obtained for TRN20-TST80. Those obtained for the 20% prediction core were slightly lower than those of the diversity core. These results indicate that the diversity and prediction core subsets of 20% size are good predictors of the remaining Iranian accessions, and could be useful for incorporating genomic prediction when introgressing exotic germplasm into elite adapted germplasm. For Iranian accessions, the analysis not accounting for population structure gave superior accuracies of models M3 and M4 over those used when accounting for population structure (Table A3, *Appendix*) and the 10% and 20% predictive core sets were as good as the 10% and 20% diversity core sets at predicting the testing set. Also, the differences in prediction accuracy between the 10% and 20% diversity and prediction core sets were small.

## Discussion

In this research, we studied genome-enabled prediction accuracy in two different collections of gene bank accessions, one Mexican and one Iranian. Prediction accuracy obtained without accounting for population structure was always higher (15–20%) than prediction accuracy obtained when accounting for population structure. However, in both cases, the prediction accuracy for highly heritable traits in wheat gene bank accessions ranged from intermediate to high. These results may stimulate the application of genomic prediction in gene bank evaluation and germplasm enhancement programs to speed up the process of introgressing diversity into elite germplasm. In general, results of this study are in agreement with the simulation study by Gorjanc *et al.* (2016) of maize genetic resources, in the sense that prebreeding can start directly from the large genetic diversity in gene bank landraces. Furthermore, the relatively high accuracies obtained from the 10% and 20% core sets (diversity and prediction) indicate that the methods used to form these core sets resulted in reference training sets that are representative of the whole collection, and can predict the remaining population fairly accurately.

### Prediction accuracy of the random cross-validation scheme *vs.* the diverse core set and the prediction core set

The TRN20:TRT80 analyses represent a random selection of lines for the training population. This set always produced higher accuracy than did the diversity and prediction core sets (Table 4 and Table 5). For traits measured in one environment, when accounting for population structure, the prediction accuracy of the 20% diversity and 20% prediction core sets were, on average, about 1.2–2.8% lower for Mexican and Iranian landraces, and almost 8.7% lower for the 20% prediction core of the Iranian collection when compared to the results of TRN20:TRT80 (Table 5). On average, the decrease in prediction accuracy of the 10% prediction core set *vs.* TRN20-TST80 was greater (10.3% and 14.9%) than the percent change in accuracy of the 20% diversity for Mexican and Iranian collections (2.7% and 2.8%). The percent change in accuracy of the 20% prediction core *vs.* TRN20-TST80 was lower than the percent change in accuracy of the 20% diversity core for the Mexican collection.

**Table 5:** Average percent change in prediction accuracy of 10% and 20% diversity and prediction cores *vs.* prediction accuracy of random cross-validation TRN20-TST80 (first four columns) and percent change in prediction accuracy between 10% diversity core *vs.* 10% prediction core, and between 20% diversity core *vs.* 20% prediction core for traits measured in one or in two environments for Mexican and Iranian collections

Trait-Collection*^a^*	10% Diversity	10% Prediction	20% Diversity	20% Prediction	10% Diversity *vs.* 10% Prediction	20% Diversity *vs.* 20% Prediction
Accounting for population structure						
One environment -Mexican collection	8.0	10.3	2.7	1.2	2.4	−1.6
One environment -Iranian collection	13.1	14.9	2.8	8.7	1.2	5.9
Two environments -Mexican collection	7.5	6.5	3.0	1.2	−1.3	−1.9
Two environments -Iranian collection	12.1	7.6	0.9	3.3	−5.3	2.4
Not accounting for population structure						
One environment -Mexican collection	4.1	4.7	0.9	0.9	0.6	−0.1
One environment -Iranian collection	7.5	25.5	1.94	9.5	1.9	19.5
Two environments -Mexican collection	2.51	2.72	0.59	0.63	0.13	0.03
Two environments -Iranian collection	8.30	5.11	0.81	1.46	−3.48	0.63

a Traits evaluated in one environment when accounting for population structure (from Table 2), traits evaluated in two environments when not accounting for population structure (from Table 4), traits evaluated in one environment when not accounting for population structure (from Table A1), traits evaluated in two environments when not accounting for population structure (from Table A3).

For traits measured in two environments (DTH and DTM), in four cases, the 10% and 20% prediction cores had a smaller reduction in their correlations with respect to the TRN20-TST80 set, than those observed for the 10% and 20% diversity core sets for the Mexican and Iranian collections (Table 5) (6.5 *vs.* 7.5 7.6 *vs.* 12.1 5.11 *vs.* 8.30 1.2 *vs.* 6.5).

The main advantage of the 10% and 20% diversity and prediction core sets over the random cross-validation is that they generate in one time a good prediction training set. The random cross-validation partitions explore and sample the entire correlation space between observed and predicted values (*i.e.*, some of these correlations are low or even negative, indicating a low training-testing relationship, while others are high, indicating a close relationship between training-testing sets), while the core sets focus directly on the subset that will produce high correlations between training-testing sets.

As discussed above, the two sizes (20% and 10%) of the Mexican and Iranian landrace core sets gave different prediction accuracies, with the 20% core set being slightly better than the 10% core set. These results confirm the hypothesis that phenotyping and genotyping a small number of accessions and genotyping the entire population can give relatively high prediction accuracies, thereby prompting the application of GS to predict the trait value gene bank accessions for traits with relatively high heritability. The results of this study indicate that one could phenotypically evaluate only 20% of germplasm bank accessions using the reference core set of accessions for GS model training and then the values of the rest of the collection. The 20% core sets of the Mexican landraces were still large (*n* > 1600), while the 20% core sets of the Iranian landraces were relatively small (*n* > 400). However, genomic prediction of traits with low heritability will likely require using training sets of greater size, and prediction accuracy would not be expected to reach values as high as those found in this study for highly heritable traits.

As breeding programs begin to implement GS, maintaining genetic variance will be increasingly important because GS has been shown to lead to faster losses of genetic variance compared to phenotypic selection (Jannink 2010; Rutkoski *et al.* 2015). Although starting with high genetic variance will not prevent its rapid loss, it will allow starting the breeding process at higher levels of allelic diversity and genetic variance while ensuring good prediction accuracies.

Breeders can use predicted values developed from the core set to identify a diverse set of accessions with desired values, and use them to start a prebreeding project. Breeders will likely select only lines with the desired trait value based on phenotypes or GEBVs to initiate a prebreeding program. For example, breeders may only select lines with the highest yield (actual or predicted). It is possible that the GS model built on the entire phenotypically and genetically diverse core set will not predict the value of the progeny from a more restricted set of selected parents. The GS model may need to be retrained with this subset of parents.

### Prediction accuracy of the diverse core sets *vs.* the prediction core sets

For genomic selection (GS), a good training set of individuals to be genotyped and phenotyped is crucial for predicting the value of candidates in the testing set. Therefore, designing a core training population that both maximizes genetic diversity and increases the accuracy of GS models is a key issue as phenotyping becomes more expensive. The method used in this study to form the diversity core was developed by Franco *et al.* (2006) using all the markers, and with the objective of maximizing allele diversity. The method proposed by Akdemir *et al.* (2015) to approximate the prediction error variance (PEV) as a reliability measure based solely on the first 100 principal components of the marker data was used to select the prediction core sets. Our results indicated very similar prediction accuracies were obtained when using the diversity *vs.* the prediction core set (Table 5). Better prediction accuracy of the 10% prediction core over the 10% diversity core was clear for the Iranian collection for the traits across two environments when accounting for population structure (–5.3), than when not accounting for population structure (–3.48). The 20% diversity core was superior to the 20% prediction core for traits in one environment (5.9 and 19.5). For the rest of the comparisons, differences in prediction accuracy between the diversity and prediction core set were negligible, ranging from –1.3 to 2.4. These results indicated similar prediction accuracies for both kinds of cores, regardless of their size.

### Diversity of the diversity and prediction core sets

Franco *et al.* (2006) described and applied three diversity indices to compare the diversity of different core sets formed using markers: the Shannon diversity index, the expected proportion of heterozygous loci, and the number of effective alleles. We applied these indices on the diversity and prediction core sets to compare their genetic diversity.

In terms of diversity measured through the Shannon Diversity Index, the expected heterozygosity (with two alleles, the maximum is 0.5) and the number of effective alleles (the maximum value is 2 for two alleles), the two types of cores gave very similar values. For example, for the Mexican collection, the expected heterozygosity was around 0.23 for the diversity cores of both sizes, and 0.22 for the prediction cores of both sizes; the number of effective alleles for both core types of collections was around 1.4, and the Shannon Diversity Index was 0.48 for the diversity core and 0.49 for the prediction core. For the Iranian collections, the expected heterozygosity was around 0.19 for both types of core sets, and the number of effective alleles was 1.30, with a Shannon Diversity Index of around 0.5. These results indicated that forming diversity and/or prediction cores gave stability to the values of the genetic distances among accessions, and thus more diverse alleles were collected in both types of cores.

### Genomic prediction under population structure

In general, when accounting for population structure, genomic prediction accuracy decreases. In animal and human populations, fitting the first few initial principal components from analysis of marker data to adjust phenotypic values is a common practice for accounting for population structure. Daetwyler *et al.* (2015) pointed out that accounting for spurious population structure (such as that originated from admixtures without affecting relatedness) is required; however, they recognized that this is not easy. Also in breeding populations, high prediction accuracy for small training populations can be caused by population structure. Sallam *et al.* (2015) argued that structure in training populations of great size could also reduce genomic prediction accuracy.

In general, population structure is important when assessing genomic prediction in stratified populations (Windhausen *et al.* 2012), especially those comprised of gene bank landrace accessions. The population structure of these landrace collections is expected to be stronger than what can be found in a standard breeding program. The first five principal components accounted for about 25% of the global molecular variance in each landrace set and were used to adjust the phenotypic data for population structure. This seems to be an acceptable number of principal components, and was also suggested by Gou *et al.* (2014). Five principal components is a reasonable number to account for population structure because using an excessive number of principal components may remove useful genetic relationships between individuals in the training and testing sets (Daetwyler *et al.* 2015).

Also, it should be mentioned that GBLUP models capture population structures and substructures within and between families because regressing phenotypic values on all marker values is equivalent to regressing the phenotypes on all principal components derived from molecular data (de los Campos *et al.* 2010; Janss *et al.* 2012). The different degrees of genetic similarities and dissimilarities between landrace accessions could also be the result of a combination of different factors such as population structure, substructure, and subtle complex relationships due to additive × additive interaction (epistasis); these factors are captured by marker differences and consequently accounted for by genomic regression GBLUP models (Crossa *et al.* 2013).

In a recent article, de los Campos *et al.* (2015) developed quantitative genetic models that account for stratified genetic populations. Rather than dealing with stratification as a confounding effect and thus developing methods that correct for stratification, such as using marker-derived principal components as fixed covariables, these authors proposed that the approach, from a quantitative genetic perspective, should regard population structure as a modifier effect (not as a confounding effect). They argued that differences in allele frequency between marker-derived groups (population structure) induce heterogeneity of allele substitution effects at different loci. Also, different linkage disequilibrium between markers and QTL may cause heterogeneity of marker effects (population structure).

### Germplasm enhancement of wheat: the way forward

Our results show that for the two populations of landraces included in this study, genomic predictions were generally of a magnitude that could be very useful for predicting the value of other accessions in the gene bank, and that could be useful in breeding. This occurred despite pronounced population structure and G × E. The first application of this approach would be to predict the value of all genotyped accessions in a gene bank, and then phenotype those that have the highest predicted value to verify their value in breeding. Once their value has been verified, a breeder could begin prebreeding following several strategies. A major decision would be whether to initiate a prebreeding population by crossing among the accessions themselves or whether to cross the chosen accessions to elite materials. The former is a conversion approach (improve the value of exotic germplasm until it becomes elite), while the latter is an introgression approach. The genetic values modeled within the landrace populations are only relevant within those populations, and thus would only be useful in a conversion strategy. Using an introgression strategy will require crossing the best accessions to elite materials, and developing a new model of the genetic effects that would be used to predict the value of future progeny. Gorjanc *et al.* (2016) compared these strategies after cycles of simulated selection and found that crossing only among landraces (conversion) maintained the most diversity (relative to the elite gene base), but produced a low rate of improvement of genetic merit (relative to the elite base). In contrast, the introgression strategy (crossing landraces with elites followed by selection) retained less diversity after cycles of selection but produced greater genetic merit relative to the elite base, and a greater rate of gain. Their results did show that genomic selection was effective at improving genetic merit with either strategy.

Further research is required to clarify several aspects of using genomic selection in prebreeding. Work is underway at CIMMYT to do multiple introgressions of a large number of landraces into elite materials with a large number of parents and progenies that have been initially genotyped and phenotyped. This will provide excellent populations to explore the use of genomic prediction for capturing the value present in gene banks.

### Conclusions

Results of this study indicate that genomic-enabled prediction of large wheat gene bank collections comprising Mexican and Iranian landraces for highly heritable traits may be a valuable tool for germplasm enhancement. Prediction accuracies based on random cross-validation partitions of traits related to wheat grain quality measured in one environment were intermediate to relatively high (from 0.4 to 0.6) for Mexican (7 traits) and Iranian (8 traits) landraces. Reference diversity and prediction core sets of sizes 10% and 20% of the total number of accessions gave good prediction accuracy, especially cores of size 20% that achieved similar prediction accuracies as those obtained by random cross-validation set (TRT20-TST80). Core sets of 10% had a substantial decrease in prediction accuracy (up to 16%) as compared with the prediction accuracy of the 20% core sets.

Traits DTH and DTM were evaluated in drought and heat environments. Genomic models including genomic × environment (G × E) interaction gave substantial and consistent increases in prediction accuracy over the main effect models though the accession × environment interaction did not account for much variability. For Mexican landraces, models including G × E had the highest prediction accuracies, reaching correlations of 0.59–0.60 for both traits when 80% or 90% of the total number of accessions were predicted across environments when accounting for population structure. Iranian landraces had similar but slightly lower prediction accuracies. When not accounting for population structure, traits DTH and DTM for a large number of Mexican accessions were predicted with accuracies of 0.7–0.75.

This study used extensive wheat landrace data stored in the CIMMYT gene bank, and has shown promising results in terms of prediction accuracies of highly heritable traits that should stimulate further research on utilizing gene bank accessions with high-density markers for future application of genomic prediction in germplasm enhancement programs.

## Appendix

**Table A1:** Not accounting for population structure

Trait	TRN20-TST80	10% Diversity Core	20% Diversity Core	10% Prediction Core	20% Prediction Core
Mexican landraces					
Plant height (PHT)	0.451 (0.006)	0.409	0.463	0.401	0.454
Thousand-kernel weight (TKW)	0.767 (0.004)	0.747	0.756	0.752	0.752
Test weight (TW)	0.687 (0.004)	0.668	0.677	0.671	0.687
Grain hardness (GH)	0.617 (0.006)	0.586	0.614	0.596	0.613
Grain protein (GP)	0.733 (0.004)	0.715	0.729	0.710	0.729
SDS sedimentation (SDS)	0.599 (0.006)	0.573	0.570	0.563	0.584
Grain yield per square meter (GYSM)	0.570 (0.005)	0.556	0.568	0.540	0.564
Iranian landraces					
Plant height (PHT)	0.260 (0.023)	0.207	0.271	0.1540	0.224
Thousand-kernel weight (TKW)	0.598 (0.014)	0.560	0.557	0.4751	0.548
Test weight (TW)	0.567 (0.013)	0.532	0.554	0.3790	0.534
Grain hardness (GH)	0.618 (0.013)	0.575	0.610	0.4628	0.522
Grain protein (GP)	0.493 (0.017)	0.497	0.484	0.3864	0.471
Grain length (GL)	0.683 (0.013)	0.626	0.667	0.6281	0.626
Grain width (GW)	0.688 (0.012)	0.645	0.669	0.4432	0.645
SDS sedimentation (SDS)	0.456 (0.017)	0.427	0.445	0.3689	0.3938

Mean correlation between predicted and observed values across 30 random cross-validation partitions (SD in parentheses) for a training set of 20% (TRN20) and a testing set of 80% (TST80), of the total Mexican and Iranian collections for several traits measured in a single environment using the GBLUP model. Correlation using 10% diversity and prediction cores and 20% diversity and prediction cores as training sets to predict the remaining 90% and 80% of the collections.

**Table A2:** Not accounting for population structure in Mexican and Iranian landraces

	Estimated Variance Component						Percentage of Within-Environment Variance				
	E	A	G	A × E	G × E	Res.	A	G	A × E	G × E	Res.
Mexican landrace											
	Days to Heading										
M1:E + A	1.127	0.051				0.038	57.35				42.64
M2:E + A + G	0.804	0.004	0.015			0.033	8.15	28.87			62.97
M3:E + A + G + G × E	0.680	0.006	0.013		0.013	0.016	13.05	27.20		26.54	33.18
M4:E + A + G + G × E + A × E	0.625	0.005	0.014	0.005	0.011	0.012	11.74	29.05	10.71	23.60	24.88
	Days to Maturity										
M1:E + A	1.034	0.230				0.106	68.39				31.60
M2:E + A + G	0.711	0.012	0.071			0.102	6.88	38.16			54.95
M3:E + A + G + G × E	0.587	0.032	0.061		0.036	0.046	18.16	34.78		20.79	26.24
M4:E + A + G + G × E + A × E	0.531	0.030	0.064	0.013	0.034	0.034	17.29	36.35	7.75	19.20	19.39
Iranian landraces											
		Days to Heading									
M1:E + A	1.075	0.038				0.041	48.35				51.65
M2:E + A + G	0.821	0.008	0.021			0.036	12.00	32.74			55.26
M3:E + A + G + GE	0.727	0.008	0.019		0.018	0.015	13.41	32.13		29.99	24.47
M4:E + A + G + G × E + A × E	0.649	0.007	0.020	0.006	0.015	0.013	11.65	32.84	9.47	24.59	21.44
		Days to Maturity									
M1:E + A	1.055	0.064				0.138	31.64				68.36
M2:E + A + G	0.778	0.012	0.043			0.118	7.11	24.57			68.14
M3:E + A + G + GE	0.686	0.014	0.032		0.067	0.043	8.93	20.46		42.80	27.81
M4:E + A + G + G × E + A × E	0.603	0.012	0.034	0.015	0.058	0.035	7.82	21.91	9.94	37.38	22.95

Estimated variance components for four models and percentage of within-environment variance accounted for by each random effect of the corresponding model using the full data for two traits, days to heading (DTH) and days to maturity (DTM). E, environment; A, accession; G, genomic (marker); A × E, accession × environment; G × E, genomic × environment; Res., residual.

**Table A3:** Not accounting for population structure in Mexican and Iranian landraces

Trait	Model*^a^*	TRN20-TST80	10% Diversity Core	20% Diversity Core	10% Prediction Core	20% Prediction Core
Mexican collection						
DTH	M1:E + A	0.000 (0.010)	−0.012	0.013	−0.009	0.001
	M2:E + A + G	0.696 (0.004)	0.676	0.692	0.677	0.689
	M3:E + A + G + G × E	0.738 (0.004)	0.718	0.732	0.718	0.735
	M4:E + A + G + G × E + A × E	0.739 (0.004)	0.717	0.732	0.720	0.735
DTM	M1:E + A	−0.002 (0.009)	−0.016	0.009	−0.004	0.005
	M2:E + A + G	0.737 (0.003)	0.721	0.735	0.717	0.733
	M3:E + A + G + G × E	0.762 (0.003)	0.745	0.759	0.740	0.757
	M4:E + A + G + G × E + A × E	0.762 (0.003)	0.746	0.758	0.742	0.756
Iranian collection						
DTH	M1:E + A	−0.003 (0.021)	0.015	0.008	−0.015	0.019
	M2:E + A + G	0.525 (0.088)	0.475	0.521	0.503	0.516
	M3:E + A + G + G × E	0.600 (0.056)	0.552	0.600	0.553	0.577
	M4:E + A + G + G × E + A × E	0.598 (0.052)	0.544	0.591	0.558	0.581
DTM	M1:E + A	−0.001 (0.023)	0.0035	−0.0086	0.000	0.009
	M2:E + A + G	0.469 (0.106)	0.4396	0.4557	0.469	0.475
	M3:E + A + G + G × E	0.587 (0.032)	0.5406	0.5889	0.549	0.581
	M4:E + A + G + G × E + A × E	0.587 (0.030)	0.5337	0.5841	0.556	0.584

Mean correlation across 30 random partitions between observed and predicted values of four models for two traits, days to heading (DTH) and days to maturity (DTM), across two environments (standard deviation in parentheses), for 20% training (TRN20) and 80% testing (TST80) sets of the total number of accessions in the Mexican and Iranian collections for four models (M1–M4). Correlations between observed and predictive values for 10% and 20% diversity and prediction core sets.

a Models: E, Environment; A, accession; G, genomic relationship; A × E, accession × environment interaction; G × E, genomic × environment interaction.
